# Evaluation of podiatric disorders in a sample of children with intellectual disabilities: an analytical cross-sectional study

**DOI:** 10.1590/1516-3180.2018.0202161118

**Published:** 2018-12-13

**Authors:** Laura Cala-Pérez, Marta Elena Losa-Iglesias, David Rodríguez-Sanz, César Calvo-Lobo, Daniel López-López, Ricardo Becerro-de-Bengoa-Vallejo

**Affiliations:** I DP, MSc, PhD. External Collaborator, Department of Nursing, Faculty of Health Sciences, Universidad Rey Juan Carlos, Madrid, Spain.; II RN, DP, BSc, MSc, PhD. Professor, Department of Nursing, Faculty of Health Sciences, Universidad Rey Juan Carlos, Madrid, Spain.; III DP, PT, MSc, PhD. Assistant Professor, Faculty of Sport Sciences, Universidad Europea de Madrid, Madrid 28670, Spain.; IV PT, MSc, PhD. Assistant Professor, Department of Nursing and Physical Therapy, Faculty of Health Sciences, Universidad de León, Ponferrada, León, Spain.; V DP, BSc, MSc, PhD. Professor and Researcher, Health and Podiatry Research Unit, Department of Health Sciences, Faculty of Nursing and Podiatry, Universidade da Coruña, Spain.; VI PhD. Full Professor, School of Nursing, Physiotherapy and Podiatry, Universidad Complutense de Madrid, Spain.

**Keywords:** Disabled children, Foot, Foot injuries, Intellectual disability, Musculoskeletal disease

## Abstract

**BACKGROUND:**

Intellectual disabilities (IDs) usually derive from neurodevelopmental disabilities. They limit intellectual functioning and cause adaptive behaviors and orthopedic problems. These disabilities have harmful effects on health, everyday practical skills and social functioning, and they diminish quality of life. The goal of our research was to perform podiatric evaluations on schoolchildren with and without ID and ascertain their records of foot disorders.

**DESIGN AND SETTING:**

Analytical cross-sectional study conducted at a podiatric clinic in the city of Piedras Blancas, province of Asturias, Spain.

**METHODS:**

An analytical cross-sectional study on 82 schoolchildren affected by ID, compared with 117 healthy schoolchildren, was conducted at a podiatric clinic. Demographic data, clinical characteristics and measurements relating to podiatric examinations were recorded among the participants who completed all phases of the tool that was used in the study process.

**RESULTS:**

Almost 90% of the schoolchildren with and without ID presented foot disorders relating to smaller toes, nail disorders, flat feet or lower-limb alterations.

**CONCLUSIONS:**

The participants showed elevated prevalence of foot disorders. Podiatric evaluations are a significant means for preventing the appearance of medical conditions and/or foot problems, and they also improve general health.

## INTRODUCTION

Intellectual disabilities (IDs) are a grouping of developmental diseases characterized by impairment of cognitive functions. They give rise to learning difficulties, adaptive behavior and diminished abilities.^1^ The prevalence of ID is around 1-2% in countries of various income levels.^2^^,^^3^ Presence of IDs has been correlated with a myriad of conditions, including genetic syndromes,^4^ genitourinary system diseases, physical health problems,^5^ psychiatric alterations,^6^ seizure disorders^7^ and foot problems,^8^ among others.^9^

Currently, over 72% of people with IDs present foot disorders. However, management of these individuals’ foot health is often ignored, underestimated or neglected. These individuals tend not to have good access to foot care through regular podiatric examinations.^10^ Moreover, foot conditions may have a negative impact on overall health. These individuals may present higher incidence of orthopedic foot surgery, relating to pathological conditions of greater severity.^11^^,^^12^

Furthermore, foot disorders are associated with fatigue, difﬁculties in walking,^13^ postural problems^14^ and foot pain. These conditions affect people both with and without ID regarding their activities of daily life.^15^ The value of taking IDs into account is recognized by clinicians and healthcare policymakers,^6^ given the high incidence of foot disorders.^8^ Nonetheless, the prevalence of foot alterations and disorders in the ID population is unknown because of the scarcity of epidemiological studies. Hence, early diagnosis and control over foot disorders, general disorders and musculoskeletal conditions, and avoidance of use of inappropriate shoes, have important beneﬁts for overall health, social functioning and mobility among people with IDs.^8^

## OBJECTIVE

The goal of our study was to perform podiatric evaluations on schoolchildren with and without ID and to compare their records of foot disorders. We hypothesized that schoolchildren both with and without ID would show presence of increased prevalence of foot conditions, in this period of life of the school-age population.

## METHODS

### Participants

This was an analytical cross-sectional study conducted at a podiatric clinic in the city of Piedras Blancas, province of Asturias, Spain, between January 2013 and January 2015. A non-randomized and consecutive sampling method was used to select schoolchildren who were affected by ID, according to the Diagnostic and Statistical Manual of Mental Disorders (DSM-5),^16^ in comparison with healthy schoolchildren. The sample size calculation is described below.

Three follow-up visits were made during the period of this study: after one year (first follow-up visit in 2013), after two years (second follow-up visit in 2014) and after three years (third follow-up visit in 2015). The parents and/or legal guardians of these children provided informed consent for them to take part in the study.

The inclusion criteria for cases in this study were that they needed to present deficits in intellectual functioning and adaptive functioning that were registered in their historical medical records, in accordance with the criteria of the Diagnostic and Statistical Manual of Mental Disorders,^17^ but without any clinical signs of dementia, or any previous surgery or other signiﬁcant orthopedic treatments. The subjects that participated as controls had no register of ID or medical problems in their medical records.

The exclusion criteria included the following situations: previous history of cardiovascular disease, being immunocompromised, having suffered foot trauma or undergone previous foot surgery, having major orthopedic malformations, presence of a neurological condition, being non-autonomous or semi-autonomous in daily activities, refusal of parents to sign the informed consent form and incapacity to comprehend the instructions relating to the investigation and/or carry them out. Controls were matched to cases, in conformity with their ages.

### Procedure

At enrolment, anthropometric measurements were made, data were collected and a detailed podiatric examination was performed, always by the same trained podiatric evaluator. Thefirst step, before any measurements were made, was to record information regarding the subjects’ general health status, demographic characteristics, gender, age and history of injuries, at their visit to the podiatric clinic. This was done for all participants, with or without ID. After participants had been found to be eligible for inclusion in the study, their anthropometric data were collected. The body mass index (BMI) was calculated from the subjects’ weight in kilograms, divided by the square of their height in meters, i.e. BMI = weight/height^2^ (kg/m^2^).^18^

The participants were then divided into two groups of schoolchildren: with and without ID, matched according to age. Theytook off their shoes and socks for podiatric examinations to be conducted. These examinations followed the protocol described by Concolino etal.,^19^ using a Waldrop scale,^20^ in which a single researcher assessed the legs using a LED podoscope device (Herbitas.com, Polígono Industrial, Foios, Valencia, Spain). For this appraisal, each child stood barefoot in a bipodal relaxed posture, with feet slightly apart and weight evenly distributed. Static and dynamic examinations were performed to enable detection of biomechanical abnormalities over various periods of time in the gait cycle. This evaluator could not be blind to the subject’s ID.

Joint hyperlaxity was then evaluated. The range of foot movement was assessed in terms of abduction, adduction, dorsiflexion, plantar flexion, muscle tone (analyzed through testing manual counter-resistance), integrity of the ankle (evaluated through a drawer test) and patellar subluxation. Moreover, the foot examination involved recognition of the general appearance of the feet, abnormalities of the toes, condition of the toenails, rotation of the feet, presence of arches, foot type and skin pathology.^19^

#### Sample size calculation

The sample frame was analyzed using the clinical epidemiology research software of the University of Coruña (http://www.fisterra.com/mbe/investiga/9muestras/9muestras2.asp). The statistical treatment was based on schoolchildren living in the city of Piedras Blancas, in the province of Asturias, northwestern Spain, where the total number of schoolchildren on January 1, 2013, was 123,833 (http://www.ine.es/jaxiT3/Datos.htm?t=9681).

The sample size calculation assumed the following: a two-tailed test, an alpha level of 0.05, a desired power analysis level of 90% with a beta level of 5%, a precision of 3% for a proportion of 50% (P = 0.5) and a loss of schoolchildren of 15%. From this, it was determined that at least 156 subjects would need to be analyzed.

### Ethics considerations

This study protocol was reviewed and approved by the local research ethics committee (Universidad Rey Juan Carlos, Spain), under registration number 050520165316, with the application date of June 24, 2016. All the parents and/or legal guardians of the participating children signed a written informed consent statement before these participants with or without ID were brought into the study.

### Statistical analysis

Demographic and clinical characteristics were analyzed, including the participants’ heights, weights, ages and BMI. Thequantitative variables were summarized as means and standard deviations (SD), and maximum and minimum values, and comparisons were made between the two groups (with or without ID). Categoricalvariables were shown as total values and percentages.

All the variables were examined for normality of distribution using the Kolmogorov-Smirnov test.Independent Student t tests were used to find out whether differences were statistically significant when normal distribution was shown. Measurements that were not normally distributed were tested using the nonparametric Mann-Whitney U test. Fisher’s exact test was used to compare categorical qualitative variables.

In all of the analyses, the statistical significance level was established as P < 0.05. All the analyses were performed using a statistical software package (SPSS 19.0, Chicago, IL, USA).

## RESULTS

### Sample characteristics

A total of 197 children could be included in this study: 80 with ID who were capable of understanding the instructions necessary to carry out the present study; and 117 in the control group. All 197individuals completed all the stages of the study process: 107 were male (54.31%) and 90 were female (45.68%). Their ages ranged from 4 to 15 years and their mean age was 8.76 ± 2.33 years.

Table 1 shows the descriptive statistics on successive evaluations of age and anthropometric data. Only the variables of weight, height and BMI had normal distribution. The age of the group of participants with disabilities was significantly greater than that of the control group at the beginning of the study, as indicated by the corresponding Student t test for two independent samples. Forthis reason, the initial weight of the group with disability was significantly greater than that of the control group. In addition, the initial height of the group with disability was greater and the BMI of the group of children with disabilities was also greater than that of the control group.

**Table 1.** Sociodemographic and clinical characteristics of the sample population

Variable	Total group Mean ± SD (range) N = 197	ID group Mean ± SD (range) N = 80	Control group Mean ± SD (range) N = 117	P-value
Age (years)	8.76 ± 2.33 (4.0-15.0)	9.1 ± 3.08 (4.0-15.0)	8.52 ± 1.61 (6.0-11.0)	0.092**
Weight (kg)	35.04 ± 13.62 (16.60-85.30)	39.39 ± 17.58 (16.6-85.3)	32.1 ± 9.0 (17.7-56.4)	< 0.001*
Height (cm)	134.73 ± 13.61 (108.70-180.10)	136.90 ± 17.30 (108.2-180.1)	133.25 ± 10.17 (112.0-155.0)	0.064*
BMI (kg/m^2^)	18.64 ± 3.99 (11.16-32.50)	19.93 ± 4.82 (11.16-32.50)	17.76 ± 3.03 (12.16-26.12)	< 0.001*

ID = intellectual disabilities; BMI = body mass index; SD = standard deviation. In all the analyses, P < 0.05 (with a 95% confidence interval) was considered statistically significant. P-values are from an independent Student t test* or a nonparametric Mann-Whitney U test**.

Overall, 89.84% of the participants (n = 177) stated that they had suffered from foot problems. Subsequent physical examination revealed that all of them presented non-neutral calcaneal stance, 69 (38.98%) had hallux deformities, 85 (48.02%) had metatarsus adductus and 52 (28.37%) had lower limb pain.

The frequencies of foot and leg pathological conditions, comprising hallux deformities, wide spacing between the 1^st^ and 2^nd^ toes, abnormalities of the 3^rd^, 4^th^ and 5^th^ toes, flat foot and lower-limb pain, were greater in the group with intellectual disabilities than in the control group, as shown in Table 2.

**Table 2.** Frequency of foot and leg pathological conditions in the sample population

	Total group N = 197 n (%)	ID group N = 80 n (%)	Control group N = 117 n (%)	P-value
Relaxed calcaneal stance (left foot not neutral)	177 (89.84%)	70 (87.6%)	107 (91.45%)	0.472
Relaxed calcaneal stance position (right foot not neutral)	169 (85.78%)	67 (83.75%)	102 (87.5%)	0.537
Hallux deformities	69 (34.67%)	38 (46.3%)	31 (26.5%)	0.006
Wide space between 1^st^ and 2^nd^ toes	52 (26.39%)	14 (17.5%)	38 (35.8%)	0.021
Deep crease between 1^st^ and 2^nd^	32 (16.08%)	15 (18.5%)	17 (16.0%)	0.438
Abnormalities of 2^nd^ toe (leftfoot)	20 (10.15%)	9 (11.25%)	11 (9.4%)	0.810
Abnormalities of 2^nd^ toe (rightfoot)	21 (11.65%)	12 (15.0%)	9 (7.8%)	0.156
Abnormalities of 3^rd^ toe (leftfoot)	30 (15.07%)	20 (24.4%)	10 (8.5%)	0.002
Abnormalities of 3^rd^ toe (rightfoot)	33 (16.58%)	21 (25.6%)	12 (10.3%)	0.005
Abnormalities of 4^th^ toe (leftfoot)	45 (22.84%)	24 (30.0%)	21 (17.9%)	0.057
Abnormalities of 4^th^ toe (rightfoot)	49 (24.62%)	28 (34.1%)	21 (17.9%)	0.007
Abnormalities of 5^th^ toe (leftfoot)	48 (24.12%)	25 (30.5%)	23 (19.7%)	0.066
Abnormalities of 5^th^ toe (rightfoot)	49 (24.87%)	26 (32.5%)	23 (19.7%)	0.045
2^nd^ toe longer than 1^st^ toe	42 (21.31%)	14 (17.5%)	28 (23.9%)	0.294
Metatarsus adductus (left foot)	83 (42.13%)	27 (33.75%)	55 (47.0%)	0.077
Metatarsus adductus (rightfoot)	84 (42.63%)	33 (41.25%)	51 (43.6%)	0.770
Flatfoot (left foot)	22 (11.05%)	19 (23.5%)	3 (2.6%)	0.001
Flatfoot (right foot)	17 (8.54%)	14 (17.3%)	3 (2.6%)	0.001
Negative foot progression angle	25 (12.56%)	9 (11.3%)	16 (13.7%)	0.668
Lower limb pain	52 (26.13%)	12 (26.7%)	40 (30.2%)	0.003

Fisher’s exact test was performed. In all the analyses, P < 0.05 was considered statistically significant with a 95% confidence interval.

The data relating to the range of motion of the foot are shown in Table 3. The dorsiflexion values for the ankle with knee flexed or extended, inversion, eversion and dorsiflexion of the first metatarsophalangeal joint were similar between the two groups. Theplantarflexion values for the ankle were higher in the control group, but the group with intellectual disability had a greater range of plantar flexion of the 1^st^ metatarsophalangeal joint.

**Table 3.** Range of motion of foot and leg: joint pathological conditions in the sample population

	Total group Mean ± SD (range) N = 197	ID group Mean ± SD (range) N = 80	Control group Mean ± SD (range) N = 117	P-value
Left ankle dorsiflexion (knee extended)	14.32 ± 4.48 (0.0-28.0)	14.5 ± 5.3 (0.0-27.0)	14.2 ± 3.8 (3.0-28.0)	0.246
Right ankle dorsiflexion (knee extended)	14.33 ± 4.54 (0.0-30.0)	14.5 ± 5.4 (0.0-30.0)	14.2 ± 3.9 (2.0-26.0)	0.237
Left ankle dorsiflexion (knee flexed)	19.26 ± 4.53 (0.0-33.0)	19.4 ± 5.5 (0.0-32.0)	19.1 ± 3.7 (7.0-33.0)	0.485
Right ankle dorsiflexion (knee flexed)	19.35 ± 4.51 (0.0-35.0)	19.7 ± 5.5 (0.0-35.0)	19.1 ± 3.7 (5.0-31.0)	0.114
Left ankle plantarflexion	54.34 ± 8.18 (0.0-81.0)	52.9 ± 10.3 (0.0-81.0)	55.4 ± 6.1 (45.0-74.0)	0.038
Right ankle plantarflexion	16.27 ± 2.98 (4.0-27.0)	53.4 ± 9.9 (5.0-27.0)	56.2 ± 6.1 (4.0-24.0)	0.027
Eversion (left foot)	17.03 ± 3.28 (0.0-30.0)	17.2 ± 4.2 (0.0-30.0)	16.9 ± 2.5 (5.0-24.0)	0.886
Eversion (right foot)	16.84 ± 3.11 (0.0-28.0)	17.0 ± 3.7 (0.0-28.0)	16.7 ± 2.6 (4.0-25.0)	0.664
Inversion (left foot)	37.46 ± 3.69 (5.0-51.0)	37.3 ± 5.0 (5.0-47.0)	37.6 ± 2.5 (30.0-51.0)	0.818
Inversion (right foot)	37.51 ± 3.15 (20.0-50.0)	37.5 ± 4.0 (20.0-49.0)	37.6 ± 2.5 (33.0-50.0)	0.606
Plantarflexion of 1^st^ metatarsophalangeal joint (left foot)	45.87 ± 3.45 (30.0-67.0)	46.3 ± 4.0 (30.0-67.0)	45.6 ± 3.0 (35.0-63.0)	0.016
Plantarflexion of 1^st^ metatarsophalangeal joint (right foot)	45.98 ± 3.47 (30.0-68.0)	46.3 ± 3.9 (30.0-65.0)	45.8 ± 3.2 (35.0-68.0)	0.050
Dorsiflexion of 1^st^ metatarsophalangeal joint (left foot)	77.43 ± 10.07 (20.0-93.0)	77.3 ± 11.2 (20.0-93.0)	77.5 ± 9.2 (40.0-92.0)	0.599
Dorsiflexion of 1^st^ metatarsophalangeal joint (right foot)	77.59 ± 10.14 (15.0-93.0)	77.2 ± 11.7 (15.0-93.0)	77.8 ± 9.0 (44.0-93.0)	0.506
Relaxed calcaneal stance position (grades) (left foot)	4.74 ± 3.26 (-5.0-13.0)	4.7 ± 3.7 (-5.0-13.0)	4.8 ± 2.9 (-2.0-12.0)	0.932
Relaxed calcaneal stance position (grades) (right foot)	3.85 ± 3.11 (-5.0-12.0)	4.2 ± 3.6 (-3.0-12.0)	3.6 ± 2.8 (-5.0-12.0)	0.616
Chippaux-Smirak index (left foot)	34.53 ± 17.66 (0.0-84.0)	40.7 ± 19.1 (0.0-84.0)	30.2 ± 15.3 (0.0-73.0)	< 0.001
Chippaux-Smirak index (right foot)	37.28 ± 18.55 (0.0-90.0)	43.3 ± 19.4 (7.0-90.0)	33.1 ± 16.8 (0.0-81.0)	< 0.001
Lower limb length discrepancy (mm)	8.4 ± 6.50 (3.0-30.0)	14.0 ± 9.5 (5.0-30.0)	6.0 ± 2.5 (3.0-10.0)	0.062

ID = intellectual disabilities; SD = standard deviation. In all the analyses, P < 0.05 (with a 95% confidence interval) was considered statistically significant. P-values are from Mann-Whitney U test.

No significant differences were observed between the two groups regarding the degree of relaxed calcaneal stance or discrepancy between the lower limbs. However, the Chippaux-Smirak index values were found to be greater in the group with intellectual disabilities than in the control group, and ankle plantarflexion was less in the group with intellectual disabilities than in the control group.

## DISCUSSION

We conducted a cross-sectional study consisting of podiatric evaluations on schoolchildren with and without ID and ascertained their records of foot disorders. Today, in Spain, schoolchildren generally do not have good access to foot care through regular podiatric examinations, even in situations in which foot alterations and disorders are present. These are often ignored because of the existence of other major complicating diseases.

Most previous studies on this issue have addressed detection of foot problems in children with ID.^19^^,^^21^^,^^22^ However, to our knowledge, there are no studies demonstrating that careful podiatric examination among schoolchildren with and without ID shows higher incidence of foot disorders during this period of life of the school-age population.

Our research demonstrated that careful podiatric examination during the school-age period showed elevated incidence of foot conditions. Most of the anomalies that we found may have been secondary to hypotonia and laxity of the muscles and ligaments.^19^^,^^23^ This was similar to the findings from other studies that have investigated footproblems, and it suggests that the most critical problems are based on other, less common orthopedic abnormalities.^24^^,^^25^

There are important variations relating to the morphology and function of the lower extremities during the school-age period. These contribute towards changes relating to postural sway, variations in plantar loading patterns during gait and presence of flatter feet or greater pronation in the foot, with higher prevalence of bunions, pain, muscle weakness and smaller-toe alterations, increased plantar pressure and difficulty in putting shoes on.^26^

This study had some limitations that need to be acknowledged. Firstly, this investigation was conducted at a podiatric clinic with a relatively small number of subjects. Secondly, a larger and more diverse sample (including children with ID in different countries) would be beneficial, to improve the strength of the study and make it possible to identify more of the mechanisms involved. Thirdly,there was only a single non-blinded evaluator analyzing the participants’ feet. Future studies should have at least two blinded evaluators: one evaluator to examine the alterations and deformities of the feet and another to manage the disorders of the lower limbs, in comparison with the blinded data that has been recorded. Despite the existence of obvious demographic differences between the ID and control groups, future studies should consider using normalized demographic data in order to compare the group with ID with a matched paired control group.

The issues highlighted above show that there is a need for further continuous research on this trend, in order to analyze different foot conditions and the therapeutic interventions that physicians could use to improve foot health during the school-age period.

## CONCLUSION

Our study showed that clinical signs such as hallux deformities, abnormalities of the third, fourth and fifth toes, flat feet, limited range of motion for ankle plantarflexion and for first metatarsophalangeal joint and higher Chippaux-Smirak index (i.e. showing flat feet) were clinical characteristics with higher prevalence among children with intellectual disabilities, compared with a control group.
